# Rheological Properties and Modification Mechanism of Emulsified Asphalt Modified with Waterborne Epoxy/Polyurethan Composite

**DOI:** 10.3390/ma17215361

**Published:** 2024-11-01

**Authors:** Maorong Li, Zhaoyi He, Jiahao Yu, Le Yu, Zuzhen Shen, Lin Kong

**Affiliations:** 1 School of Civil Engineering, Chongqing Jiaotong University, Chongqing 400074, China; yjhcqjtu@163.com (J.Y.); yulecqjtu@163.com (L.Y.); shenzuzhencqjtu@163.com (Z.S.); 2 National and Local Joint Engineering Laboratory of Traffic Civil Engineering Materials, Chongqing Jiaotong University, Chongqing 400074, China; 3 School of Civil Engineering, Southwest Jiaotong University, Chengdu 610031, China; konglinmyswjtu@163.com

**Keywords:** waterborne epoxy, waterborne polyurethane, emulsified asphalt, response surface design, modification mechanism

## Abstract

In research aimed at improving the brittleness of WER (waterborne epoxy)-modified emulsified asphalt, commonly encountered issues are that the low-temperature performance of this type of asphalt becomes insufficient and the long curing time leads to low early strength. Matrix-emulsified asphalt was modified with WPU (waterborne polyurethane), WER, and DMP-30 (accelerator). Firstly, the performance changes of modified emulsified asphalt at different single-factor dosages were explored through conventional performance tests and assessments of its adhesion, tensile properties, and curing time. Secondly, based on a response surface methodology test design, the material composition of the composite-modified emulsified asphalt was optimized, and its rheological properties were analyzed by a DSR test and a force–ductility test. Finally, the modification mechanism was explored by scanning electron microscopy (SEM) and Fourier transform infrared spectroscopy (FTIR). The results show that WER can improve the adhesion strength of modified emulsified asphalt and greatly reduce elongation at break. WPU can effectively improve the elongation at break of composite-modified emulsified asphalt, but it has a negative impact on adhesion strength. DMP-30 mainly affects the curing time of modified emulsified asphalt; EPD (composite modification) can effectively improve the high-temperature rutting resistance of matrix-emulsified asphalt, and its low-temperature performance is significantly improved compared with WER-modified emulsified asphalt. The EPD modification process mainly consists of physical blending. In the case of increasing the curing rate, it is recommended that the contents of WER and WPU be lower than 10% and 6%, respectively, to achieve excellent comprehensive performance of the composite modification.

## 1. Introduction

Due to the multiple coupling effects of loading and the environment, asphalt pavement is subject to early diseases, such as cracking and deterioration, in the course of use [1,2,3]. The preventive maintenance of asphalt pavement refers to the proactive maintenance measures taken to delay the excessive decay of pavement performance when asphalt pavement has good performance but has minor diseases [4,5,6]. Selecting appropriate maintenance techniques and materials during the preventive maintenance stage can significantly reduce the incidence of pavement diseases, extend the service life of asphalt pavement, and greatly reduce maintenance costs, which is in line with the concept of global green transportation development [7,8,9]. Emulsified asphalt (EA) is widely used in preventive maintenance due to its good construction and anisotropy. However, the low adhesion and early strength of ordinary emulsified asphalt cannot meet the performance requirements imposed by traffic volume and environmental impact [10,11,12]. Therefore, the development of modified emulsified asphalt with excellent performance has become a research hotspot in the field of asphalt pavement maintenance.

Adding polymer modifiers is one of the most effective methods for optimizing the performance of emulsified asphalt [13,14,15]. Waterborne epoxy resin (WER), as a common polymer, becomes strong mainly due to the curing reaction between a waterborne epoxy emulsion and the curing agent and has excellent mechanical properties [16,17,18,19]. Wang et al. [20] prepared modified emulsified asphalt with different contents of waterborne epoxy resin and studied its high- and low-temperature performance and bonding performance through macro- and micro-experiments. It was found that the addition of waterborne epoxy resin significantly improved the high-temperature performance and bonding performance of modified emulsified asphalt, but its low-temperature performance deteriorated. Yang et al. [21] prepared waterborne epoxy-modified emulsified asphalt by using both a two-step and a one-step method. It was found that when the WER content was about 15%, the continuous phase of the system transformed from emulsified asphalt to WER, and WER had a certain adverse effect on the low-temperature performance of emulsified asphalt. He et al. [22] studied the adhesion and high-temperature creep recovery properties of waterborne epoxy resin (WER)-emulsified asphalt and found that when the WER content was 15%, the irreversible creep compliance of WEREA (waterborne epoxy-emulsified asphalt) tended to be stable. Shi et al. [23] introduced the acrylic monomer MAA into waterborne epoxy resin for grafting to enhance the bonding performance of modified emulsified asphalt. It was found that when the amount of waterborne epoxy resin exceeded 40%, the tensile strength of waterborne epoxy resin-modified emulsified asphalt was significantly improved at 40 °C. When the content reached 50%, its storage stability peaked. However, at present, WER-modified emulsified asphalt still has the problems of high brittleness, insufficient low-temperature performance, and the delayed opening of traffic after curing [24,25,26].

Waterborne polyurethane (WPU) is synthesized via the polycondensation of isocyanates, soft segment polyols, hydrophilic chain extenders, etc. Soft segment oligomer polyols cause it to have excellent flexibility at low temperatures [27,28,29,30]. At the same time, WPU has good compatibility with waterborne epoxy resin and emulsified asphalt due to its water solubility and can form a three-dimensional interpenetrating network structure with WER [31,32,33], further improving the mechanical properties of the system. Therefore, the addition of WPU can improve the road performance of asphalt binder [34], but there is still a lack of research and analyses on the synergistic modification and toughening of emulsified asphalt by waterborne epoxy/polyurethane/accelerator, and most existing evaluation methods are basically aimed at the asphalt system [35]. The rheological properties and microscopic modification mechanism of the waterborne epoxy resin/polyurethane system are rarely reported.

In this study, the accelerator 2,4,6-tris (dimethylaminomethyl) phenol (DMP-30) was introduced to optimize the curing reaction of WER. At the same time, WPU was introduced to improve the low-temperature toughness of emulsified asphalt. The effective components of the composite modification (EPD) were epoxy resin/polyurethane/accelerator. The rheological properties and modification mechanism of WER-, WPU-, and EPD-composite-modified emulsified asphalt were studied by a conventional performance test, a response surface methodology test design, a dynamic shear rheological (DSR) test, a scanning electron microscope (SEM), and an infrared spectroscopy test. Compared with the current conventional single modification, this study combines the practical problems in the application of emulsified asphalt and innovatively introduces waterborne epoxy resin, polyurethane, and accelerator to modify traditional emulsified asphalt. From the perspective of composite material optimization and modification, the material’s own performance advantages are fully considered in order to improve the flexibility and low-temperature performance of WER-modified emulsified asphalt, thereby preventing pavement cracking and prolonging its service life.

## 2. Materials and Methods

### 2.1. Raw Materials

The waterborne epoxy emulsion used in the test was bisphenol A-type epoxy resin, the curing agent was triethylenetetramine (TETA), and industry-grade waterborne polyurethane (WPU) was provided by Anhui Anda Huatai New Materials Co., Ltd., Hefei, China. The accelerator 2,4,6-tris (dimethylaminomethyl) phenol (DMP-30) was provided by Samson New Materials Co., Ltd., Shanghai, China. The associated technical indicators are shown in Table 1 and Table 2. Emulsified asphalt is a self-made cationic slow-cracking-type compound, and the relevant technical indicators are shown in Table 3. Among them, the softening point of emulsified asphalt evaporation residue refers to the temperature at which asphalt softens and deforms under heating conditions and is expressed in °C.

### 2.2. Preparation Method and Process of Modified Emulsified Asphalt

**Figure 1:**
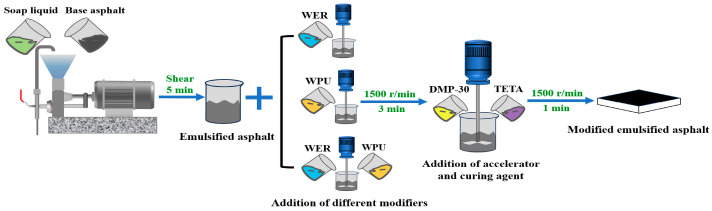
Schematic diagram of modified emulsified asphalt preparation.

Waterborne epoxy emulsion with mass fractions of 5%, 10%, and 15% was added to the emulsified asphalt, and a high-speed shearing machine was used for cutting at 1500 rpm for 3 min to make it uniformly dispersed. Then, the curing agent triethylenetetramine with a mass fraction of 60% of waterborne epoxy emulsion was added to the mixture of waterborne epoxy emulsion and emulsified asphalt, and the modified emulsified asphalt with different WER contents was prepared by shearing at 1500 rpm for 1 min in a high-speed shearing machine. The preparation method of WER/WPU (contents of 3%, 6%, and 9%) composite-modified emulsified asphalt was the same, in which DMP-30 (contents of 2%, 3%, and 4%) was added prior to the curing agent, and the preparation process is shown in Figure 1.

### 2.3. Experimental Methods

#### 2.3.1. Conventional Physical Test

(1) Three Indicators Test

The evaporation residue was prepared, and the three indexes of evaporation residue of different modified emulsified asphalt specimens were tested according to ASTM D5 [36], ASTM D36 [37], and ASTM D113 [38].

(2) Adhesion Test

The spraying amount of modified emulsified asphalt coated on a 100 mm × 100 mm × 30 mm cement block was 0.8 g/cm^2^, and the adhesion test was carried out after curing at 25 °C.

(3) Tensile Property Test

The tensile test was carried out according to the tensile test method in GB/T 16777:2008 [39].

(4) Curing Time Test

In order to characterize the curing speed of modified emulsified asphalt, the test temperature was set to 40 °C to accelerate its curing, and 5 g of emulsified asphalt was poured into the evaporation dish. At the beginning of curing, it was weighed every 10 min. When the mass deficit reached 60%, it was measured every 5 min. When the mass difference between the two residues was less than 1%, it was recorded as the complete curing time.

#### 2.3.2. Response Surface Experimental Design

Based on the Box–Behnken theory, a three-factor and three-level response surface test was designed, and elongation at break, curing time, and adhesion strength were used as output indicators to determine the optimal content of each component. The single-factor dosage design was as follows: the WER dosages were 5%, 10%, and 15%; the WPU contents were 3%, 6%, and 9%; the DMP-30 contents were 2%, 3%, and 4%. The Box–Behnken response surface test design was implemented by using Design-Expert 11 software.

#### 2.3.3. High-Temperature Performance Test

The phase angle (*δ*) and complex modulus (G*) of modified emulsified asphalt were measured by a dynamic shear rheological (DSR) test. The thickness of the sample was 1 mm, the diameter was 25 mm, the initial temperature was 46 °C, the temperature gradient was 6 °C, the scanning frequency was 10 rad/s, and the termination condition rut factor was G*/*sinδ* ≤ 1.0 KPa. At the same time, the multi-stress creep mode (MSCR) was used to test the high-temperature rheological properties. The stress levels were 0.1 kPa and 3.2 kPa, and each stress level was subjected to 10 cycles. The load shear creep lasted 1 s, and the unloading recovery lasted 9 s. The high-temperature rheological properties were studied by using the average elastic recovery rate *R* and the average irreversible creep compliance *J_nr_*. The specific formulas are shown in Equations (1) and (2).

(1) $$R=\frac{\varepsilon_{m}-\varepsilon_{nr}}{\varepsilon_{m}-\varepsilon_{0}}\times 100\%$$

(2) $$J_{nr}=\frac{\varepsilon_{nr}-\varepsilon_{0}}{\tau }\times 100\%$$

where $\varepsilon_{0}$ is the initial strain of each creep recovery cycle, $\varepsilon_{m}$ and $\varepsilon_{nr}$ are the peak strain and irrecoverable strain of each creep recovery cycle, respectively, and *τ* represents the shear stress, kPa.

#### 2.3.4. Low-Temperature Performance Test

**Figure 2:**
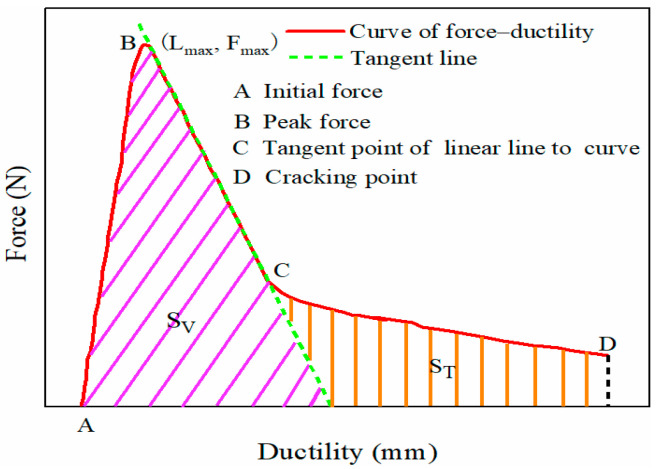
Schematic diagram of force–ductility.

The temperature of the force–ductility test was 10 °C, the tensile speed was 1 cm/min, and the test ended when the modified emulsified asphalt cracked. The low-temperature performance of composite-modified emulsified asphalt was evaluated based on the tensile flexibility *f*, and the toughness ratio *R*_*T*/*V*_ indexes [40], and the specific equations and schematic diagrams are shown in Equations (3) and (4) and Figure 2.

(3) $$f=L_{max}/F_{max}$$

(4) $$R_{T/V}=S_{T}/S_{V}$$

where $F_{max}$ and $L_{max}$ are the peak force and the corresponding ductility, and $S_{V}$ and $S_{T}$ represent the area enclosed by the tangent line to the X-axis at the descending section of the force–ductility curve and the area enclosed by the tangent line to the X-axis at the end of the force–ductility curve, respectively.

#### 2.3.5. Microstructure Analysis

The asphalt specimens were pre-sprayed with gold in a 5 kV, 5 mA vacuum diffraction coater for 8 min, and a Zeiss Sigma 300 (Oberkochen, Germany) scanning electron microscope (SEM) was used to test the fracture surface morphology of WER-, WPU-, and EPD-modified emulsified asphalt force–ductility specimens, after which their low-temperature cracking resistance and toughening mechanism were analyzed.

#### 2.3.6. Fourier Transform Infrared Spectroscopy (FTIR) Test

A Nicolet iS 10 infrared spectrometer from Thermo Fisher Scientific (Waltham, MA, USA) was used to test the change in functional groups of modified emulsified asphalt, with the experimental spectral range from 4000 cm^−1^ to 400 cm^−1^ at a resolution of 4 cm^−1^ and the scanning frequency of 32 times/s.

## 3. Results

### 3.1. Conventional Performance of Single-Factor-Modified Emulsified Asphalt

**Figure 3:**
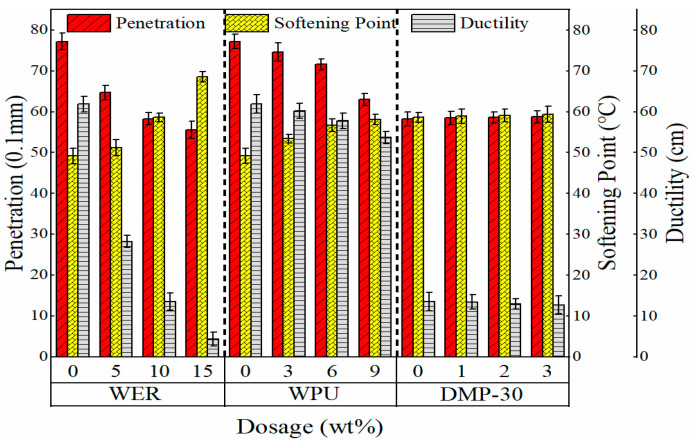
Effects of different doses of WER, WPU, and DMP-30 on penetration, softening point, and ductility.

Modified emulsified asphalt specimens with WER (contents of 0%, 5%, 10%, and 15%), WPU (contents of 0%, 3%, 6%, and 9%), and DMP-30 (content of 0%, 2%, 3%, and 4%) were prepared, and the three indicators of the evaporation residue of the modified emulsified asphalt were measured. The results are shown in Figure 3.

It can be seen from Figure 3 that the ductility and penetration of WER-modified emulsified asphalt decreased with the increase in WER content, and the ductility of 15% WER-modified emulsified asphalt was 3.3 cm, causing almost brittle fracture. The softening point of WER-modified emulsified asphalt increased with the increase in WER content, from 49.2 °C to 68.5 °C with 15% content, an increase of 39%. This is mainly because epoxy resin forms a three-dimensional network structure in asphalt. With the increase in the content, steric hindrance restricts the molecular chain movement of the light component of asphalt, which increases the viscosity of the system [21]; the stiffness modulus of WER-modified emulsified asphalt evaporation residue increased with the increase in epoxy resin content, which led to the weakening of the stress relaxation ability of emulsified asphalt, thus sharply decreasing its ductility. This is consistent with the findings of Wang et al. [20]. In comparison with the modified emulsified asphalt with 10% WER content, the penetration and softening point of asphalt with WPU at 9% content were similar, and the ductility was much better than that of WER-modified emulsified asphalt. WPU-modified emulsified asphalt had slightly lower ductility than the original emulsified asphalt, but it had better low-temperature ductility than the WER modification. This is because the WPU soft segment structure improves the low-temperature toughness, but as the content continues to increase, it also leads to the deterioration of low-temperature performance, which is caused by the aggregation of polyurethane particles at high WPU content [21]. DMP-30 was mainly used as a catalyst in the system, and its influence on the three indicators of modified asphalt was less than 1%, which did not allow us to distinguish the promoting influence of DMP-30 effectively, thus necessitating further analysis.

### 3.2. Adhesion Analysis

**Figure 4:**
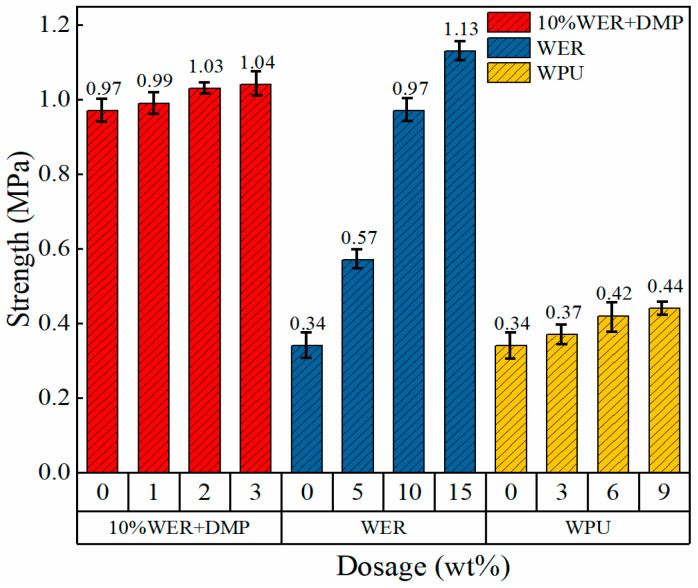
Adhesion test results of single-factor-modified emulsified asphalt.

In order to control for a single variable to analyze the modification effect of the DMP-30 accelerator, the 10% WER specimen was taken as a sample for analysis and was tested by an XH-M electric adhesion tester, and the test results are shown in Figure 4.

It can be seen from Figure 4 that the adhesion strength of the original asphalt increased by about 68% at 5% WER content, and the strength at 10% content reached 0.97 MPa, but the increase in strength was no longer significant when the WER content was 15%. WPU had little effect on the adhesion of modified emulsified asphalt, and its strength increased with the increase in WPU content. Due to the strong ductility of WPU, its own stiffness modulus to resist deformation was low, but the strength of WPU was still better than that of emulsified asphalt, thus improving adhesion to emulsified asphalt. The increase in adhesion strength of 10% WER-modified emulsified asphalt with DMP-30 content increased first and then decreased, but when the content was 3%, the increase in adhesion strength decreased from 6% to 1%, which can be attributed to the subsequent reaction of an epoxy group and amine group, due to which the apparent activation energy of the blend system reached the minimum and the strength reached the maximum level. DMP-30 is characterized by the presence of amine groups, and it is speculated that some of them may participate in the curing reaction. Therefore, increasing the amount of accelerator has little effect on the adhesion strength of the system.

### 3.3. Tensile Properties

**Figure 5:**
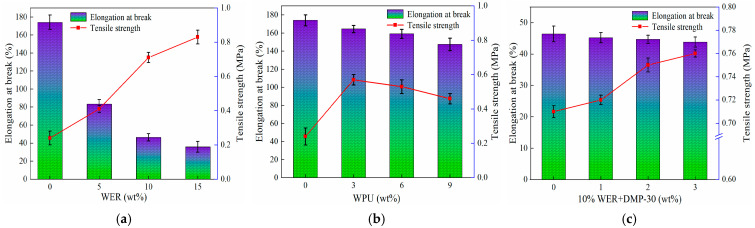
Tensile test results of different single-factor-modified emulsified asphalt: (**a**) WER; (**b**) WPU; (**c**) 10% WER + DMP-30.

A tensile test was used to determine the toughness of modified emulsified asphalt, and the performance of modified emulsified asphalt was evaluated in terms of tensile strength and elongation at break [26]; the results of the test are shown in Figure 5.

Figure 5a reveals that the increase in WER content resulted in a relative increase in the tensile strength of modified emulsified asphalt, along with a gradual decrease in elongation at break. However, when the content of WER was 5–10%, the effect on elongation at break was obvious. When the content exceeded 10%, the modification effect was significantly reduced. Due to the high stiffness modulus of WER, waterborne epoxy reacts with emulsified asphalt to form a three-dimensional network structure in the original asphalt, which improves the tensile strength. At the same time, this leads to low deformation characteristics of WER systems: elongation at break becomes worse, and toughness decreases. According to Figure 5b, the elongation at break of polyurethane-modified emulsified asphalt gradually decreased with the increase in WPU content. When the WPU content reached 3%, the tensile strength reached the maximum value, and the tensile strength began to decrease with the increase in WPU content. The main reason is that excessive WPU will lead to the aggregation of polyurethane particles, which will deteriorate the strength of modified emulsified asphalt. Given its excellent tensile properties, WPU has a less significant effect on the elongation at break of emulsified asphalt systems than WER, but its structure forms an interspersed interaction structure with asphalt, and its elongation at break is reduced. It can be seen from Figure 5c that the increase in DMP-30 content resulted in a gradual decrease in the elongation at break of 10% WER-modified emulsified asphalt, although to an insignificant extent. When the content of DMP-30 was 1% to 2%, the maximum increase in tensile strength was 4.2%. When the content was 2% to 3%, the tensile strength increased from 0.75 MPa to 0.76 MPa and continued to increase due to the significant improvement in the curing rate of the system after adding DMP-30 to the WER blending system, which completed the reaction formally. If the curing agent is further increased, its mechanical properties may be degraded.

### 3.4. Curing Time Characterization

**Figure 6:**
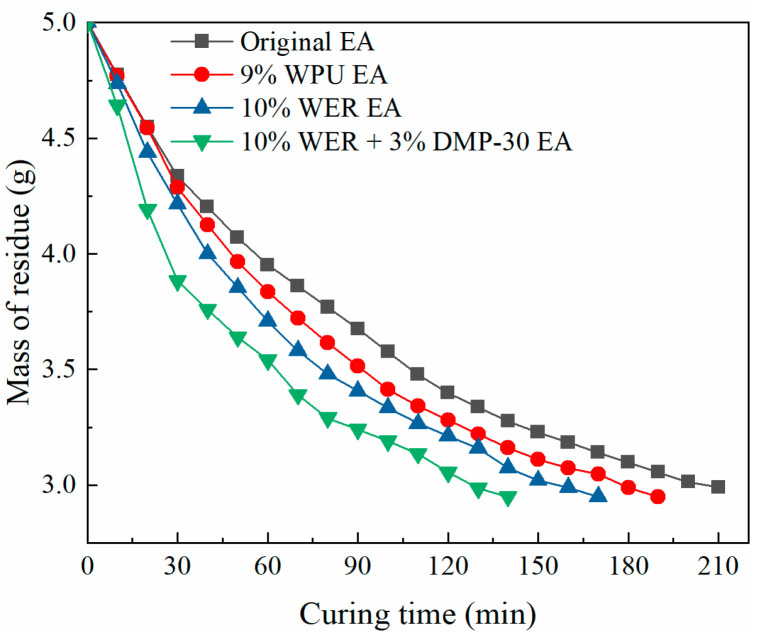
Curve of curing time–residual mass.

The curing time of emulsified asphalt with nearly 10% WER content was tested. The results of the mass loss and time curve are shown in Figure 6.

As can be seen from Figure 6, the curing speed of modified emulsified asphalt was higher than that of the base emulsified asphalt; compared with the base emulsified asphalt, the 9% WPU-modified emulsified asphalt’s curing time was shortened by 9.5%, and 10% WER-modified emulsified asphalt showed a curing time 19% shorter than the base emulsified asphalt. The main reason is that the exothermic curing reaction of WER plays a certain role in promoting curing, and the polymerization of the modifier is slightly faster than the asphalt particles, which is more conducive to water evaporation. Further, 3% DMP-30 effectively improved the curing rate of WER-modified emulsified asphalt by 18%. This is because the accelerator DMP-30 can reduce the reaction activation energy of WER systems, increase the collision probability of the amino group and epoxy group, and release more heat in a short time, thus accelerating the curing rate of WER-modified emulsified asphalt.

### 3.5. Response Surface Analysis

Composite-modified emulsified asphalt is not just a simple addition of each component. In order to determine the optimum dosage of each component of EPD composite-modified emulsified asphalt, the Box–Behnken response surface test design was implemented by using Design-Expert 11 software, and the test scheme and results are shown in Table 4.

The regression model was obtained through the regression fitting analysis of the experimental data of adhesion strength (A), elongation at break (B), and curing time (C), as shown in Equations (5)–(7).

A = −0.931 + 0.176X + 0.024Y + 0.592Z − 0.002XY − 0.001XZ + 2.875YZ − 0.005X^2^ − 0.003Y^2^ − 0.100Z^2^ (5)

B = 9.875 − 2.5X + 8.167Y + 70.75Z − 0.117XY − 0.15XZ + 0.083YZ − 0.065X^2^ − 0.403Y^2^ − 11.125Z^2^ (6)

C = 362.5 − 7.999X − 12.083Y − 62.5Z + 0.001XY + 0.5XZ − 1.352e^−15^YZ + 0.2X^2^ + 1.111Y^2^ + 7.5Z^2^ (7)

where X, Y, and Z represent the contents of WER, WPU, and DMP-30, respectively, and the corresponding content ranges are 5 wt% ≤ X ≤ 15 wt%, 3 wt% ≤ Y≤ 6 wt%, and 1 wt% ≤ Z ≤ 3 wt%.

#### 3.5.1. Model Validation and Analysis

To further ensure the validity and accuracy of the response surface model, the fitted regression model needed to be analyzed through the analysis of variance (ANOVA) test and the significance of the regression coefficients tested. ANOVA was performed on the test results of A, B, and C, and the significance was determined by the F test [41]. The results are shown in Table 5.

The significance test of the regression coefficient reveals that if the F-value is larger and the corresponding *p*-value is smaller (usually less than 0.05), it indicates that the relationship between the independent variable and the dependent variable in the model is significant and the model is reliable. It can be seen from Table 5 that the F-values of the three regression models A, B, and C are about 542.654, 128.470, and 10.320, respectively, and the corresponding *p*-values are far less than the F-values. Therefore, the model has good relevance and statistical significance; the *p*-values of each mismatch term are all greater than 0.05, indicating that the proportion of abnormal errors in the model fitting is small and the difference is not significant. In addition, the correlation coefficients *R*^2^ of the three models are 0.997, 0.929, and 0.982, respectively, which further indicates that the model is reliable and the fitting accuracy is high, which can well describe the relationship between the response values and the corresponding factors.

#### 3.5.2. Adhesion Strength

**Figure 7:**
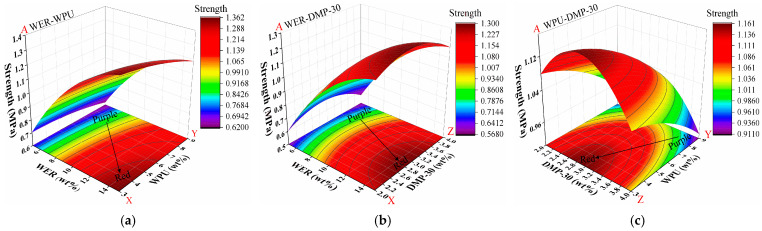
Response surface results of adhesion strength: (**a**) WER-WPU; (**b**) WER-DMP-30; (**c**) WPU-DMP-30.

The correlation coefficient of determination *R*^2^ of 0.997 was obtained by performing the quadratic fitting of the data, which showed a good fit; the results of adhesion strength (A) with WER content (X), WPU content (Y), and DMP-30 content (Z) are shown in Figure 7.

It can be seen from Figure 7a,b that under the interaction of synergistic modification, WER had the greatest influence on the adhesion strength of modified emulsified asphalt. With the increase in epoxy resin content, the adhesion strength gradually increased, showing that the greater the proximity to the right end, the more obvious the red area. The contour line density was more concentrated at 5% and 10%. When the content exceeded 10%, the contour line density gradually decreased, indicating that the speed of strength growth decreased. From Figure 7c, it can be seen that when WPU synergistically modified emulsified asphalt, the blue area gradually deepened with the increase in dosage, indicating that WPU had a negative impact on the adhesion strength of modified emulsified asphalt. When the content of WPU exceeded 6%, the contour density increased, and the strength of emulsified asphalt was greatly degraded by the high content of WPU. When the contents of the accelerator were 2% and 3%, this mainly improved the adhesion strength of emulsified asphalt, and when the content was 4%, it weakened the adhesion strength of emulsified asphalt.

#### 3.5.3. Tension Toughness

**Figure 8:**
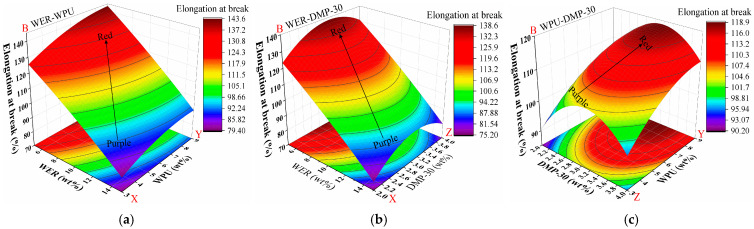
Response surface results of elongation at break: (**a**) WER-WPU; (**b**) WER-DMP-30; (**c**) WPU-DMP-30.

The results of the data were fitted, and the correlation coefficient of determination *R^2^* was 0.929, which indicates a good fit. The results of elongation at break (B) according to WER content (X), WPU content (Y), and DMP-30 content (Z) are shown in Figure 8.

From the analysis of Figure 8a,b, it can be seen that WPU had a great influence on the elongation at break of modified emulsified asphalt. With the increase in WPU content from 3% to 9%, elongation at break gradually changed from blue to red, and elongation at break decreased significantly with the increase in WPU. With the continuous increase in WPU, the density of the contour line gradually decreased, indicating that the toughening effect deteriorates with the continuous increase in WPU content. The content of WER was negatively correlated with elongation at break. With the increase in WER content, elongation at break decreased obviously. The color change in Figure 8c is small, indicating that the effect of the DMP-30 accelerator on toughness was small, and there was a slight trend of initial increase followed by a decrease.

#### 3.5.4. Curing Time 

**Figure 9:**
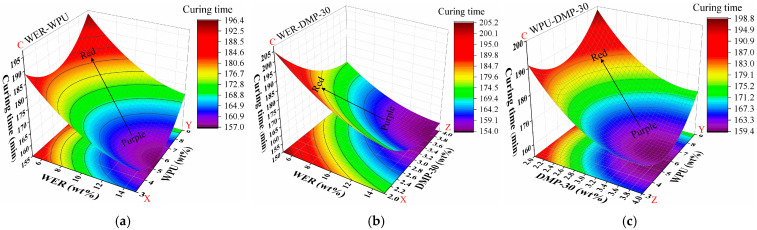
Response surface results of curing time: (**a**) WER-WPU; (**b**) WER-DMP-30; (**c**) WPU-DMP-30.

The data for curing time were fitted, and the correlation coefficient of determination *R*^2^ was 0.982, which indicates a good fit. The resultant impacts of WER content (X), WPU content (Y), and DMP-30 content (Z) on curing time (C) are shown in Figure 9.

The gradual change from a red to a purple area indicates that the curing time changed from slow to fast, and the darker color of the purple area indicates that the curing demulsification speed was higher at this dosage. The denser the contour line, the greater the influence on the curing speed. As can be seen from Figure 9, DMP-30 played a major role in influencing the curing time of modified emulsified asphalt, which was significantly reduced (see Figure 9b) as the dosage of DMP-30 increased from 2% to 4% with a gradual transition from red to blue. In Figure 9c, the continuous increase in accelerator dosage gradually decreased the contour density, indicating that the promotion effect was reduced but not reflected in the results of single-factor modified emulsified asphalt in Section 3.1, which implies that the composite modification is not a simple addition of the components. The content of WER had a strong influence on the curing time, which decreased with the increase in WER content. The WPU content had a tendency to decrease first and then increase with the curing time.

Considering the response factors of WER, WPU, and DMP-30, 10% WER exhibited excellent performance and high elongation at break. At 6% WPU content, good elongation was obtained without significantly decreasing the adhesion strength; DMP-30 as an accelerator had a significant effect on the demulsification and curing time of the modified emulsified asphalt system, and it was the best at the 3% dosage. The dosage of EPD composite modification was determined as 10% WER, 6% WPU, and 3% DMP-30. At this time, the adhesion strength was 1.11 MPa, the elongation at break was 117%, and the curing time was 160 min. The comprehensive performance of the composite modification was excellent.

### 3.6. High-Temperature Performance of Composite-Modified Emulsified Asphalt

**Figure 10:**
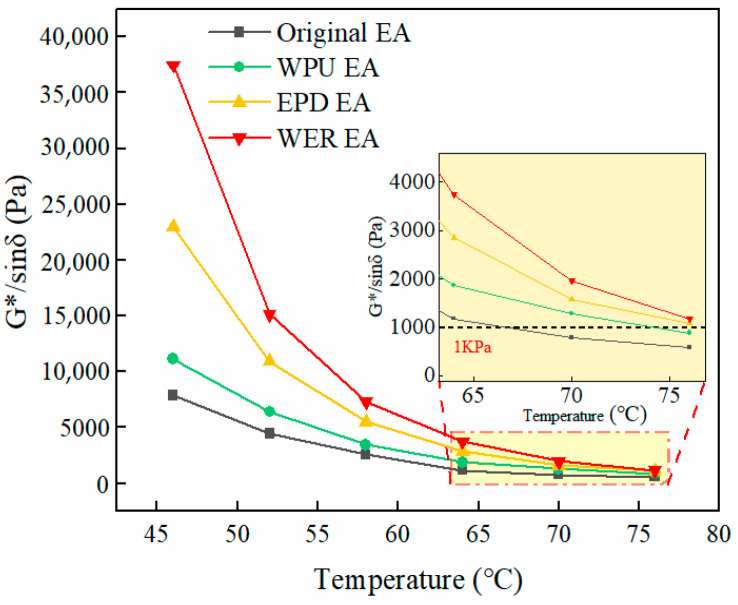
Rutting factors of various modified emulsified asphalt types.

The optimal content of WER and WPU in EPD-modified emulsified asphalt was determined to be 16% according to Section 3.5. In order to study the high-temperature rutting resistance of 16% WER and 16% WPU single-factor and EPD composite-modified emulsified asphalt specimens, the complex modulus G* and phase angle *δ* were measured in DSR temperature scanning mode, and the rutting factor G*/*sinδ* was used to characterize the ability of modified emulsified asphalt to resist permanent deformation. The test results are shown in Figure 10, wherein EA is emulsified asphalt and the corresponding acronyms represent the different types of modified emulsified asphalt. For example, WER EA is waterborne epoxy resin-modified emulsified asphalt, while Original EA is unmodified original emulsified asphalt. The acronyms appearing in the following text are also available.

It can be seen from Figure 10 that the rutting factors of WER and WPU single-component and EPD composite-modified emulsified asphalt were negatively correlated with temperature. As the temperature increases, the rutting factor gradually decreases. The rutting factor of EPD composite-modified emulsified asphalt was much higher than that of the original emulsified asphalt, indicating that the EPD composite modification can effectively improve the anti-rutting ability of the original emulsified asphalt. The main reason is that the incorporation of the EPD composite-modified material DMP-30 in emulsified asphalt makes the cross-linking reaction of the WER system more complete, and the resultant three-dimensional network structure can effectively limit the flow of emulsified asphalt at high temperatures, thus improving the performance of emulsified asphalt. The rutting factor is significantly increased, and the three-dimensional network structure can synergistically resist the load to a certain extent and improve the anti-rutting performance of emulsified asphalt. However, compared with WER-modified emulsified asphalt, the rutting factor was reduced due to the incorporation of WPU in EPD, and the stiffness modulus of WPU was low, which deteriorated high-temperature performance to a certain extent.

**Figure 11:**
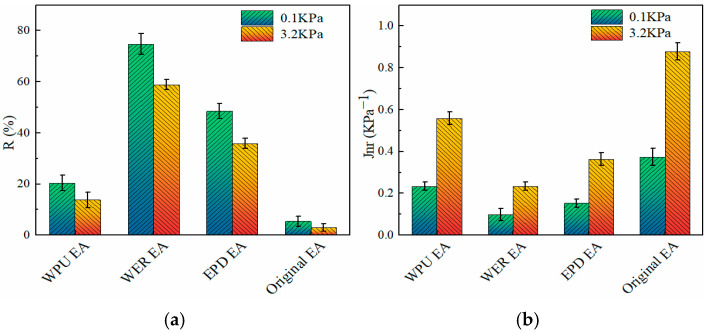
High-temperature performance parameters of modified emulsified asphalt: (**a**) creep recovery rate (*R*); (**b**) non-recoverable creep compliance (*J_nr_*).

When the temperature exceeded 70 °C, the rutting factors of different modified emulsified asphalt have a significant convergence effect. In order to further evaluate the high-temperature performance of modified emulsified asphalt accurately, the percent recovery *R* and the non-recoverable creep compliance *J_nr_* were obtained based on the multiple stress creep model (MSCR) at the stress levels of 0.1 kPa and 3.2 kPa, as shown in Figure 11.

It can be seen from Figure 11a that at the stress level of 0.1 kPa, compared with the original emulsified asphalt, the polymer modification significantly increased the creep recovery rate *R*, and the *R*-value of EPD composite-modified emulsified asphalt increased from 5% to 48%. This is mainly because the composite modification performance of EPD can effectively improve the ability of the original emulsified asphalt to resist loading and can also change the viscous system of emulsified asphalt at the same time. Due to the addition of a resin polymer, the elastic component increased relatively, thus improving the creep recovery rate significantly. At the same time, under the stress of 3.2 kPa, the creep recovery rate decreased, indicating that the high load has a greater impact on the asphalt system and is more likely to cause rutting damage to the emulsified asphalt mixture.

It can be seen from Figure 11b that the non-recoverable creep compliance *J_nr_* of EPD composite-modified emulsified asphalt was 0.41 under the stress condition of 3.2 kPa. Compared with the original emulsified asphalt *J_nr_* value of 0.87, the non-recoverable creep compliance was significantly reduced due to the three-dimensional network structure formed by the staggered distribution of EPD-modified materials in emulsified asphalt, which can effectively inhibit the fluidity of emulsified asphalt at high temperatures. When subjected to load, the strain generated is significantly reduced. At the same time, EPD increased the viscoelasticity of emulsified asphalt, which decreased the non-recoverable creep. EPD can effectively improve the high-temperature performance of matrix-emulsified asphalt.

### 3.7. Low-Temperature Performance of Composite-Modified Emulsified Asphalt

**Figure 12:**
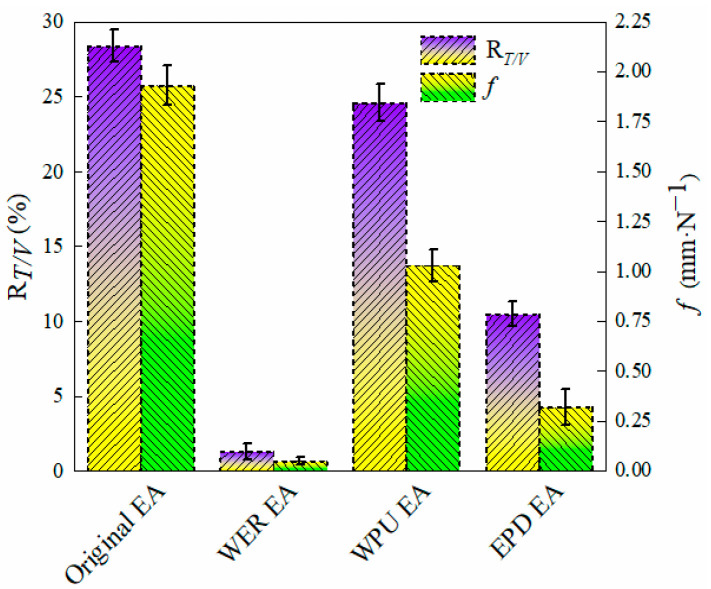
Force–ductility of different modified emulsified asphalt types.

In order to further explore the low-temperature performance of EPD-modified emulsified asphalt, original, 16% WER-, and 16% WPU-modified emulsified asphalt specimens were used as the control group. Based on the force–ductility curve, the tensile flexibility *f* and toughness ratio *R*_*T*/*V*_ were calculated to characterize the low-temperature performance of modified emulsified asphalt. The results are shown in Figure 12.

A larger toughness ratio indicates better low-temperature cracking resistance; the greater the tensile flexibility, the greater the stress relaxation capacity of asphalt and the better the low-temperature performance. From Figure 12, it can be seen that the toughness ratio *R*_*T*/*V*_ of WER-modified emulsified asphalt was 1.3%, showing a brittle fracture phenomenon, and the tensile flexibility *f* showed similar change characteristics. It is mainly due to the difficulty in making the epoxy resin molecular chain slide after WER curing, which leads to poor stress relaxation and low toughness of WER at low temperatures [42,43]. The toughness ratio *R*_*T*/*V*_ of EPD-modified emulsified asphalt was 10.5%, which is a significantly improvement compared with the low-temperature performance of WER-modified emulsified asphalt, owing to the WPU molecular chain’s richness in flexible segments composed of polyether polyols and other oligomers when the highly elastic WPU and WER are modified [44]. Therefore, WPU still has good flexibility at low temperatures. When the flexible segments of WPU overlap with the rigid segments of epoxy resin, the rigid structure changes into a flexible structure, forming a three-dimensional network structure that enhances the plastic deformation of WER emulsified asphalt. The stress relaxation ability of WER at low temperatures is improved. Therefore, the toughness ratio and tensile flexibility are greatly improved, and the low-temperature performance is significantly improved.

### 3.8. SEM Analysis

**Figure 13:**
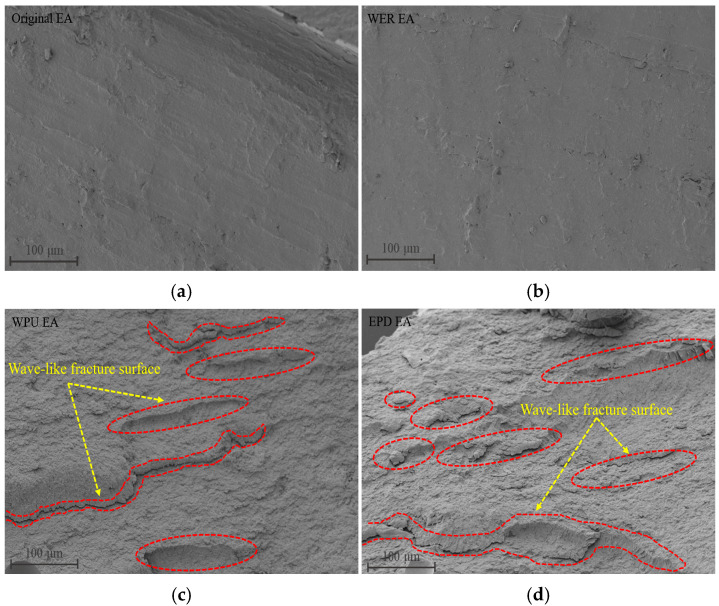
SEM images of the fracture surface of modified emulsified asphalt: (**a**) Original EA; (**b**) WER EA; (**c**) WPU EA; (**d**) EPD EA.

The fracture surfaces of different modified emulsified asphalt types obtained from the force–ductility test were subjected to SEM scanning tests, and the results are shown in Figure 13.

As seen in Figure 13a,b, the fracture surface of the WER EA specimen was smooth, which is due to the high stiffness modulus and small elastic modulus of WER emulsified asphalt after curing and the small micro-strain generated when subjected to loaded tension. Therefore, the fracture surface was smooth, and almost no deformation occurred. When subjected to tension, brittle breaks suddenly occurred at the middle position of the specimen with very small fracture elongation. From Figure 13c, it can be seen that due to the more carbamate groups on the polyurethane molecular chain in WPU emulsified asphalt, WPU emulsified asphalt exhibited the mechanical property of high elastic modulus after curing. When subjected to tensile force, the polyurethane particles in the curing system can cause better synergistic deformation of the system. The micro-strain of the system increased significantly, showing many wave-like fracture surfaces. The EPD emulsified asphalt in Figure 13d showed similar fracture surfaces due to the fact that WPU contains more flexible soft chain segments, which are intertwined with WER molecular chains to form a three-dimensional interpenetrating structure. When the EPD system is subjected to external forces, the soft segments in the WPU can disperse the stress, change the crack path, and can buffer the deformation of WER in cooperation with the load and improve the fracture elongation of the WER system. The microscopic morphology demonstrates that WPU can improve the micro-strain of WER, which is also the reason why WPU largely improves the fracture elongation of WER.

### 3.9. Infrared Spectroscopic Analysis

**Figure 14:**
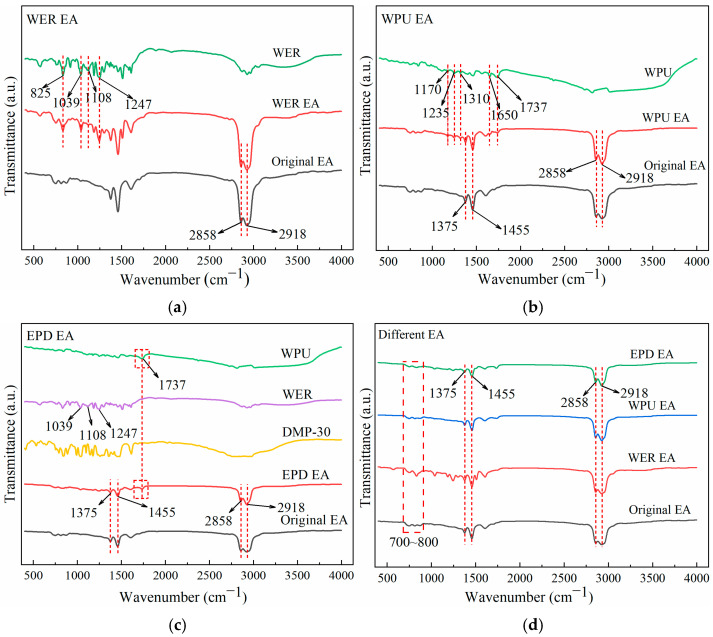
Test results of infrared spectroscopy: (**a**) WER EA; (**b**) WPU EA; (**c**) EPD EA; (**d**) different types of EA.

The infrared spectroscopy test (FTIR) was used to analyze whether new functional groups existed after modification and to investigate the modification mechanism of single-component and EPD-modified emulsified asphalt; the infrared results are shown in Figure 14.

As can be seen from Figure 14a, the main peaks of WER and emulsified asphalt are highly consistent with each other in the infrared spectral band from 2000 to 4000 cm^−1^, and the absorption peaks at 2918 cm^−1^ and 2858 cm^−1^ are caused by the asymmetric and symmetric telescopic vibration of C-H in the alkanes. The significant difference between WER-modified emulsified asphalt and the original emulsified asphalt in the 400–2000 cm^−1^ band is mainly due to the fact that the peaks of WER-modified emulsified asphalt are significantly affected by WER. The characteristic peak at 825 cm^−1^ corresponds to the epoxy group; that at 1039 cm^−1^ represents the phenyl ether functional group; the absorption peaks at 1108 cm^−1^ and 1247 cm^−1^ are caused by the C-O-C antisymmetric stretching vibration of the ether bond, and the intensity of the absorption peak is significantly enhanced. From Figure 14b, it can be seen that the WPU peak is not significant, as the peak value of WPU is not significant, so single-component-modified emulsified asphalt is easily covered by emulsified asphalt with a significant peak value. The peak fluctuation of WPU-modified emulsified asphalt is highly consistent with that of the original emulsified asphalt. The peaks at 1455 cm^−1^ and 1375 cm^−1^ are caused by the common vibration of methyl (-CH_3_) and methylene (-CH_2_), but there are peaks at 1170 cm^−1^, 1235 cm^−1^, 1310 cm^−1^, and 1650 cm^−1^ that do not appear for the original asphalt, which is basically consistent with the characteristic functional groups of WPU.

From Figure 14c, it can be seen that after EPD composite modification, due to the decrease in the relative value of WER content and the synergistic effect after composite modification, the strong fluctuation in the WER peak after EPD composite modification is weakened, but there are still slight fluctuations at 1039 cm^−1^, 1108 cm^−1^, and 1247 cm^−1^. At the same time, the characteristic peak of WPU changes significantly at 1737 cm^−1^, which is caused by the stretching vibration of carbonyl C=O in carbamate formed by chain extension reaction [34]. As shown in Figure 14d, compared with the original emulsified asphalt, the change in the characteristic peak can find the corresponding peak fluctuation in the modifier, and the change in the characteristic peak of modified emulsified asphalt is only the stacking of the peak. Due to the different dosages of the modifier, the peak changes slightly, but there is no obvious characteristic peak. Therefore, the performance improvement of EPD composite-modified emulsified asphalt mainly depends on the physical blending of each component, and the enhancement mechanism is mainly based on the polymer forming a three-dimensional network structure in emulsified asphalt.

## 4. Conclusions

(1) WPU can effectively enhance the elongation at break of EPD composite-modified emulsified asphalt, but the incorporation of WPU will reduce adhesion strength, and it is recommended that the dosage of WPU be lower than 6%.
(2) The three indicators can be used to evaluate single-factor modified emulsified asphalt, but there are some limitations in the evaluation of synergistic modification. The optimal dosage of synergistic modifier with excellent comprehensive performance can be obtained by a Box–Behnken response surface test design, and the EPD composite-modified emulsified asphalt has the most excellent comprehensive performance.
(3) The rutting factor and creep recovery rate R of EPD composite-modified emulsified asphalt are much higher than those of the original emulsified asphalt, and the unrecoverable creep compliance is significantly reduced. EPD can effectively improve the rutting resistance of matrix-emulsified asphalt.
(4) Compared with WER-modified emulsified asphalt, the low-temperature performance of EPD-modified emulsified asphalt can be improved significantly. The SEM results of the wave-like fracture surface show that WPU has better ductility and can achieve toughening modification.
(5) The infrared spectrum analysis of matrix-emulsified asphalt, WER, and WPU showed that no new substances were produced in the blending process of the EPD system, and the modification mechanism involved physical blending.
(6) In this study, the rheological properties and modification mechanism of WER, WPU, and composite-modified emulsified asphalt were studied. It was found that EPD-modified emulsified asphalt is very suitable for pavement maintenance or as a preventive maintenance binder. Future research can focus on the water damage resistance of modified emulsified asphalt and road performance under multiple coupled aging conditions and can further explain the anti-aging mechanism of WPU.

This paper proposes a technique to improve the low-temperature toughness of emulsified asphalt and provides theoretical reference and guidance for the popularization and application of waterborne epoxy/polyurethane-modified emulsified asphalt in preventive maintenance.

## Figures and Tables

**Table 1:** Basic technical indexes of waterborne epoxy emulsion and polyurethane.

Type	Appearance	Density (g/cm^3^)	Solid Content (%)	Elongation at Break (%)	pH	Viscosity (mPa·s, 25 °C)	Storage Stability (d)
WER	Milky white	1.13	52 ± 2	-	6.5–7.5	700–900	≥180
WPU	Milky white	1.06	50	1000–1400	7–9	>100	-

**Table 2:** Basic technical indexes of accelerator DMP-30 and curing agent.

Type	Appearance	Density (g/cm^3^)	Solid Content (%)	Amine Value (mgkOH/g)	Molecular Weight	Viscosity (mPa·s, 25 °C)
DMP-30	Yellowish	0.98	99.9	585–615	265	-
TETA	Brown	1.01	83	-	146.23	4000 ± 500

**Table 3:** Technical specifications of emulsified asphalt.

Test Item	Residual on Sieve (%)	Engler Viscosity (oE)	Evaporation Residue Content (%)	Evaporated Residue			Storage Stability (d)	
				Penetration (0.1 mm)	Softening Point (°C)	Ductility (cm; 15 °C)		
Result	0.02	8	60	61.4	52.4	63.7	0.6	3
Standard	≤0.1	2–30	≥60	50–130	-	≥40	≤1	≤5

**Table 4:** Box–Behnken design and results.

Test No.	WER (%)	WPU (%)	DMP-30 (%)	Adhesion (MPa)	Curing Time (min)	Elongation at Break (%)
1	5	3	3	0.69	190	126
2	15	3	3	1.37	165	78
3	5	9	3	0.61	195	145
4	15	9	3	1.20	170	90
5	5	6	2	0.61	205	122
6	15	6	2	1.21	175	76
7	5	6	4	0.58	175	130
8	15	6	4	1.16	155	81
9	10	3	2	1.08	190	91
10	10	9	2	0.95	200	104
11	10	3	4	1.04	165	96
12	10	9	4	0.91	175	110
13	10	6	3	1.12	165	115
14	10	6	3	1.13	170	118
15	10	6	3	1.12	175	112
16	10	6	3	1.11	160	117
17	10	6	3	1.11	155	113

**Table 5:** ANOVA of the fitted model.

Model	Source	Sum of Square	df	Mean Square	*F*-Value	*p*-Value	Statistical Significance	*R* ^2^
A	Model	0.911	9	0.1012	542.654	<0.0001	Significant	0.997
	Lack of fit	0.001	3	0.0003	4.881	0.0799	Not significant	
B	Model	6029.030	9	669.8900	128.470	<0.0001	Significant	0.929
	Lack of fit	10.500	3	3.5000	0.539	0.6808	Not significant	
C	Model	3481.620	9	386.8500	10.320	<0.0001	Significant	0.982
	Lack of fit	12.500	3	4.1700	0.067	0.9748	Not significant	

## Data Availability

The original contributions presented in the study are included in the article, further inquiries can be directed to the corresponding authors.
